# Call-duration and triage decisions in out of hours cooperatives with and without the use of an expert system

**DOI:** 10.1186/1471-2296-9-11

**Published:** 2008-02-13

**Authors:** Rob SG Ong, Johan Post, Harry van Rooij, Jan de Haan

**Affiliations:** 1 Cluster Zorg en Welzijn, Hogeschool Leiden, The Netherlands; 2 University Medical Centre Groningen, Netherlands, Department of General Practice, University Medical Center Groningen, Groningen, The Netherlands; 3 Coöperatie Huisartsenposten Midden-Brabant, Tilburg, The Netherlands; 4 University Medical Centre Groningen, Netherlands, Department of General Practice, University Medical Center Groningen, Groningen, The Netherlands

## Abstract

**Background:**

Cooperatives delivering out of hours care in the Netherlands are hesitant about the use of expert systems during triage. Apart from the extra costs, cooperatives are not sure that quality of triage is sufficiently enhanced by these systems and believe that call duration will be prolonged drastically. No figures about the influence of the use of an expert system during triage on call duration and triage decisions in out of hours care in the Netherlands are available.

**Methods:**

Electronically registered data concerning call duration and triage decisions were collected in two cooperatives. One in Tilburg, a cooperative in a Southern city of the Netherlands using an expert system, and one in Groningen, a cooperative in a Northern city not using an expert system. Some other relevant information about the care process was collected additionally. Data about call duration was compared using an independent sample t-test. Data about call decisions was compared using Chi Square.

**Results:**

The mean call time in the cooperative using the TAS expert system is 4.6 minutes, in the cooperative not using the expert system 3.9 minutes. A significant difference of 0.7 minutes (0.4 – 1.0, 95% CI) minutes. In the cooperative with an expert system a larger percentage of patients is handled by the assistant, patients are less often referred to a telephone consultation with the GP and are less likely to be offered a visit by the GP.

A quick interpretation of the impact of the difference in triage decisions, show that these may be large enough to support the hypothesis that longer call duration is compensated for by less contacts with the GP (by telephone or face-to-face). There is no proof, however, that these differences are caused by the use of the triage system. The larger amount of calls handled by the assistant may be partly caused by the fact that the assistants in the cooperative with an expert system more often consult the GP during triage. And it is not likely that the larger amount of home visits in Groningen can be attributed to the absence of an expert system. The expert system only offers advice whether a GP should be seen, not in which way (by consultation in the office or by home visit).

**Conclusion:**

The differences in call times between a cooperative using an expert system and a cooperative not using an expert system are small; 0.4 – 1.0 min. Differences in triage decisions were found, but it is not proven that these can be contributed to the use of an expert system.

## Background

Only a few years ago the majority of general practitioners (GPs) in the Netherlands organised the out of hours care with a few colleagues from their neighbourhood. Nowadays more than 95% of the practitioners provide out of hours care within some kind of cooperative with 50 or more other GPs. Along with this change, the way triage is performed for different types of medical aid during out of hours care has also altered. In the traditional setting triage was often handled by the GP himself, now specially trained assistants answer the phone and perform triage. The safety and quality of this form of triage is a concern to the Dutch Association of General Practitioners (LHV) as well as to those considering the issue in other countries [1-3]. Several instruments to optimize safety and quality of care are being developed; training methods for performing high quality triage are under construction, triage protocols have been developed and expert systems constructed. The use of protocols and algorithms are believed to enhance the quality and safety of telephone triage [4-8]. Expert systems which enhance their systematic use have been developed and used [6,9]. The enthusiasm of Dutch GP's to use an expert system during out of hours care has been tempered by the fact that there is no hard evidence about the improved quality of such triage and because estimated duration of calls mentioned by suppliers of these systems are considered to be very long. Call durations varying from 4 to 10 minutes were predicted and call duration without an expert system was believed to be far shorter. Users and suppliers of expert systems suggest, however that any additional telephone time during triage when using an expert system, might be compensated elsewhere in the process by more efficient triage-decisions, i.e. a larger proportion of calls handled by the assistants. A finding that is supported in literature [6,9,10].

No reliable figures comparing the duration of calls and triage-decisions in Dutch out of hours care settings (with and without the use of an expert system) are available, however. To gain more insight into call duration, triage decisions and the way they affect the total care time, answers were sought to the following questions:

• "Is there a difference between call duration of a cooperative using an expert system and one not using it?"

• "Is there a difference in triage decisions between a cooperative using an expert system and one not using it?"

## Methods

**Figure 1:**
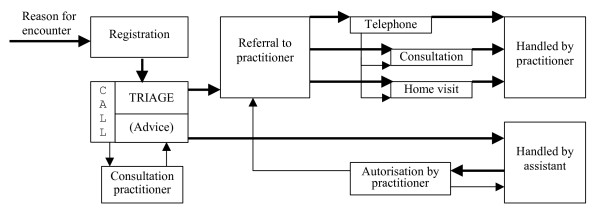
Care process Dutch cooperative.

A schematic representation of the Dutch care process during after hours care is given in figure 1. When a patient calls, name, address and insurance (NAI) records are registered first. Then the assistant will perform triage, which has 4 possible outcomes:

• (Assistant-)Advice: when the assistant handled a call. Advice is given immediately after triage, during the same call. A log recording the triage-specifics (questions asked and answers given) is made afterwards, and within a few hours a GP will check the triage decision and authorise it if he/she agrees. If the GP disagrees with the triage decision, the patient will be called back and informed.

• Telephone advice GP (TAG): when the GP handles a call by telephone and no consultation at the office or home visit follows the call in the same shift.

• Consultation at the office – when the patient is invited to visit the centre.

• Home visit – when the patient is offered a home visit.

Sometimes assistants will consult a GP during the call if they are unsure about a decision.

To be able to compare call durations and triage decisions, electronically registered data were extracted from the databases of two cooperatives. Call duration and triage decisions were automatically monitored in the cooperative of Tilburg by using the expert system. The other cooperative was equipped with a call management system that does not generate protocols to enhance decisions, but did monitor call durations and was used by the assistants to register their decisions. The data from October 2002 were extracted from the expert- and call management system of the cooperatives. All call durations were rounded off to the nearest minute as the call management system Groningen did so. All calls between 00.00 AM and 8.00 AM were excluded, as the care process in one of the cooperatives changes at night and the time spend on NAI registration is no longer separately registered from triage duration. In both cooperatives calls concerning administrative matters were excluded.

In the cooperative using the expert system a small amount of calls may not have been included, because the expert system was skipped. Sometimes when emergencies occur and direct action is clearly needed, the expert system is not used. Since emergencies do not often occur, assistants felt safe to assume that the percentage of calls during which the expert system was skipped was definitely less than 5%.

In order to gain some insight in the influence of triage decisions on total care duration, more information has been gathered about the distribution of patients among the different routes of the care process. Electronically registered triage decisions were also extracted from the expert and call management system. Furthermore, assistants and/or practitioners were asked to register the following actions for one week on specific forms:

• Number of calls in which a practitioner was consulted by the assistant,

• a. The number of the GP's telephone consultations that led to a consultation at the centre or a home visit.

• b. The number of calls handled by the assistant that were not approved by the practitioner after authorization (and the way in which they were handled afterwards: by telephone advice by the GP, by consultation at the office or home visit).

### Statistics

Mean call duration was compared by an independent sample t-test (P < 0.05). Differences in percentages of triage decisions were tested using Chi Square (p < 0.05).

### Description of cooperatives

Data from two cooperatives in the Netherlands that register duration of calls and triage decisions were extracted. At the time Tilburg, a city situated in the south of the Netherlands, had the only cooperative in the Netherlands using an expert system (nowadays there is one other city using an expert system). This system is called TAS (telephone advice system) and supports the assistant in making the right triage decision by showing the important triage-questions the assistant needs to ask and offering a triage advice based on the answers gathered. The Tilburg cooperative provides the town itself and a few small satellite villages with out of hours care: a total population of 250,000 inhabitants. The satellite villages are in close proximity to the main town and the total of their populations are very small compared to the population of the main town. Registration of name, address, insurance (NAI) and triage are performed by the same assistant, but call duration data for these actions are separately stored by the system.

Groningen (a city situated in the north of the Netherlands) has one of the few cooperatives in the Netherlands equipped with a call management system (Adastra), which registers the duration of calls and triage decisions electronically as in TAS. No triage questions or advice is offered by this system however. The Groningen cooperative services the main town of Groningen and several smaller and rural towns in the province. The total population of Groningen consists of 170,000 inhabitants. Different assistants performed registration of the NAI and triage data. To keep the populations similar (i.e. mainly urban), only the data of Groningen city were analysed.

## Results

As shown in table 1, the cooperative of Tilburg handled more calls in October 2002 than the cooperative of Groningen. The number of calls per 1000 inhabitants, however, is more or less the same. In Tilburg 16.0 contacts per 1000 occurred, in Groningen 16.3. The percentage of contacts that took place between 00.00 and 8.00 AM is similar in both cooperatives, 16%.

**Table 1:** Contacts in October 2002.

	**Tilburg**	**Groningen**
**Number of contacts between 00.00 and 08.00 h**	787	483
**Number of contacts between 08.00 and 24.00 h**	3466	2028

### Call Duration

The mean call-duration in Tilburg is on average 0.7 minute (CI: 0,4–1,0 min.) longer than the mean call-time in Groningen (see table 2). The larger percentage of calls shorter than 1 minute in Groningen contributes to this fact. It is not clear what kind of calls are shorter than 1 minute. In both cooperatives 50% of the calls are handled within 3.5 minutes. 95% are handled within 12 minutes in Tilburg and within 11 in Groningen.

**Table 2:** Call duration (calls between 08.00 u and 24.00 u).

	**Tilburg**	**Groningen**
**Mean call time (all contacts)**	4,6 min	3,9 min
**Number (%) calls with duration <= 1 min.**	32 (0,9%)	278 (13,7%)
**Number (%) call with duration 1–2 min.**	1167 (34%)	539 (27%)
**Number (%) call with duration 3–25 min.**	2265 (65%)	1208 (59,3%)
**Number (%) call with duration >25 min.**	2 (0%)	3 (0%)
**Percentiles:**		
• **50% of calls handled**	within 3,5 min.	within 3,5 min.
• **75% of calls handled**	within 6,5 min.	within 5,5 min.
• **95% of calls handled**	within 11,5 min.	within 10,5 min.

### Triage decisions and distribution of patients among various routes of the care process

In table 3 the percentages of the various routes through the process are displayed. The percentage of calls in which the practitioner was consulted by the assistant during triage varied between Tilburg and Groningen. In Tilburg consultation occurred in 21% of the calls, in Groningen in 4%.

**Table 3:** Percentages of the possible routes through the process.

	**Tilburg**	**Groningen**
	Oct 2002	Oct 2002
**% nurse consultation of practitioner during triage**	21% (171/818*)	4% (16/488*)
**Triage decisions:**		
**% handled by assistant**	49%	39%
**% handled by practitioner:**		
• **telephone advice GP**	6%	13%
• **consultation**	37%	35%
• **home visit**	8%	14%
**% handled by practitioner after authorization**	1% (3/313*)	---
**% telephone advice by practitioner resulting in a consultation at the office or home visit**	33% (5/15*)	31% (175/517)

* As registration in Groningen and/or Tilburg was incomplete on some days during the registration week, only the days on which data collection was flawless were included in the analyses.

Although percentages of triage decisions significantly differ between Groningen en Tilburg, the outcomes in both cooperatives have a similar outline: a large percentage of calls is handled by the assistant and if a patient is referred to the GP, this is done by a consultation at the centre most of the time. Telephone advice and home visits are less common.

In both cooperatives the percentage of patients contacted by the practitioner after authorization is either very small or absent. The percentage of the practitioners' telephone advice leading to face-to-face contact with a GP (i.e. consultation at the centre or home visit) is almost the same in both cooperatives.

## Discussion

This study provided actual figures concerning call duration and triage outcomes of a cooperative with and without the use of an expert system in the Netherlands. Apart from call-duration and triage decisions the complete outline of the out of hours care process in Dutch cooperatives has been drawn and all the distribution percentages among the different routes of care have been measured.

Mean call duration in the cooperative using the expert system is 4.6 minutes, 0.7 minute (95% of a minute CI: 0,4–1,0 min.) longer than in the cooperative where no expert system is used. A larger percentage of calls in Tilburg is handled by the assistant, less patients are offered TAG and home visits. It is uncertain, however, if the differences in TAG and/or home visits are caused by the use of the expert system.

The larger percentage of home visits in Groningen is not an effect of the expert system, since the expert system only assists in determining the urgency and necessity of personal contact with a GP. Whether a home visit is appropriate or if the patient should be able to come to the centre, is independently decided by the assistant or GP.

The difference in TAG between Groningen and Tilburg may well be caused by a different organisation of the care-process. In Tilburg the assistants consult the practitioner during triage in 24% of the calls, whereas in Groningen this occurs in 4%. It is possible that in Tilburg a considerably higher percentage of the patients would have been referred to TAG if there had not been the opportunity of consultation. In that case the smaller percentage of TAG in Tilburg is a result of a different organisation of the process, not an effect of the use of an expert system.

An important confounding factor influencing call-duration is the morbidity of the visiting population. If for example a lot of patients contact the centre with questions or problems that need extensive advice, call time would probably increase dramatically. Extensive advice increases mean call duration, since not only triage but also advice is included in the call time. It is unknown if the mean duration of the advice was similar in the two cooperatives, since there was no separate registration of triage and advice.

Due to the large number of calls it is no surprise that some significant differences in triage decisions were found. It is more interesting though if these differences are large enough to influence the care process: is it possible that longer call-durations in Tilburg are compensated by more efficient care process through a larger percentage of patients handled by the assistant?

To get an impression of the influence of triage decisions on the care process and total care duration, the figures presented above can be put into a simple model to calculate an estimation of the total care duration per 100 calls. The model has been derived from the example of the care process in fig.1 and divides total care duration into "assistant time" and "GP time". The mean call-duration from table 2 and the percentages of the possible routes through the process from table 3 have been used to calculate total care duration. Some choices in the model may need some explanation:

• Since the assistant answers all calls, mean call duration is multiplied by 100 (regardless of the percentage that is handled by the assistant in a cooperative).

• Percentages of telephone advice by the practitioner in table 3 only represents the contacts ended this way. They do not include the contacts followed by a consultation at the centre or a home visit. Since approximately one third of the practitioners' telephone advice leads to a consultation or a home visit, some consultations and home visits were preceded by telephone advice. These should be added to the percentage of telephone advice in table 3; therefore the percentages in table 3 have been multiplied by 1.5.

• Table 3 shows that in Groningen a larger percentage of home visits is made than in Tilburg. As explained before, this effect is not caused by the use of an expert system, however. As home visits are very time consuming, their influence on the total care time is relatively large. To correct for the influence of a different policy in home visits, the same ratio "consultation at the centre: home visits" was applied to both cooperatives. In the model Tilburgs' ratio, 37:8 (= 4,6) has been taken as reference ratio, and the ratio of Groningen has been adjusted. This has led to the distribution of 40:9 (= 4,4).

Most care durations in the model are based on estimations, only the call duration was actually measured. The mean duration of telephone advice from the GP (TAG) was derived from electronically monitored durations in Groningen. It was assumed that they would be the same in Tilburg. Ten minutes for a consultation at the office is a widely accepted estimation in the Netherlands and therefore used in the model as well. Table 4 shows that the total care duration for 100 calls is longer in Groningen, although call duration by the assistant are shorter. The longer total care duration is caused by a higher percentage of telephone advice by the GP (TAG) and a higher percentage of consultations/home visits.

**Table 4:** Calculating an estimation of total care time for handling 100 calls.

	**Tilburg**			**Groningen**		
	**% of calls**	**Estimate time**	% * time	**% of calls**	**Estimate time**	% * time

**Assistant**	100%	4.6 min	= 460 min	100%	3.9 min	= 390 min
						
**GP: TAG**	9%	6 min	= 54 min	19%	6 min	= 114 min
**Consultation at office**	37%	10 min	= 370 min	40%	10 min	= 400 min
**Home visit**	8%	20 min	= 160 min	9%	20 min	= 180 min
**Consult. during triage**	21%	1 min	= 21 min	4%	1 min	= 4 min

**Total**			605 min			689 min

**Total care time (Ass. + GP)**	1065 min			1088 min		

Although results indicate that promising differences may occur, comparing two cooperatives is a minimum. The comparison of more cooperatives would show more reliable results. Since Tilburg was the only cooperative in the Netherlands using an expert system, this is was not possible.

## Conclusion

Differences in call duration between the cooperative in Tilburg, where an expert system is used and the one in Groningen, which does not use one, are small. There is a difference in the mean call time of 0.4 – 1.0 (95% CI) minutes, and especially the percentage of calls handled within 1 minute differs.

Differences in triage decisions were found. When using the expert system a larger percentage of calls is handled by the assistant. It remains to be seen, however, if the differences in triage decisions are an effect of the expert system.

## Competing interests

The author(s) declare that they have no competing interests.

## Authors' contributions

RO designed and coordinated the study, performed data analysis and wrote draft versions of the manuscript. JP carried out data collection, advised in the interpretation of data and content of the manuscript. HvR carried out data collection, advised in the interpretation of data and content of the manuscript. JdH supervised the design, data analysis and content of the manuscript. All authors read and approved the final manuscript.

## Pre-publication history

The pre-publication history for this paper can be accessed here:


